# A simulation-based comparison of drug-drug interaction signal detection methods

**DOI:** 10.1371/journal.pone.0300268

**Published:** 2024-04-17

**Authors:** Dagyeom Jung, Inkyung Jung

**Affiliations:** Division of Biostatistics, Department of Biomedical Systems Informatics, Yonsei University College of Medicine, Seoul, Korea; University of Colorado Denver Skaggs School of Pharmacy and Pharmaceutical Sciences, UNITED STATES

## Abstract

Several statistical methods have been proposed to detect adverse drug reactions induced by taking two drugs together. These suspected adverse drug reactions can be discovered through post-market drug safety surveillance, which mainly relies on spontaneous reporting system database. Most previous studies have applied statistical models to real world data, but it is not clear which method outperforms the others. We aimed to assess the performance of various detection methods by implementing simulations under various conditions. We reviewed proposed approaches to detect signals indicating drug-drug interactions (DDIs) including the Ω shrinkage measure, the chi-square statistic, the proportional reporting ratio, the concomitant signal score, the additive model and the multiplicative model. Under various scenarios, we conducted a simulation study to examine the performances of the methods. We also applied the methods to Korea Adverse Event Reporting System (KAERS) data. Of the six methods considered in the simulation study, the Ω shrinkage measure and the chi-square statistic with threshold = 2 had higher sensitivity for detecting the true signals than the other methods in most scenarios while controlling the false positive rate below 0.05. When applied to the KAERS data, the two methods detected one known DDI for QT prolongation and one unknown (suspected) DDI for hyperkalemia. The performance of various signal detection methods for DDI may vary. It is recommended to use several methods together, rather than just one, to make a reasonable decision.

## 1. Introduction

Polypharmacy, the use of multiple medicines has increased as the average life expectancy and the prevalence of multimorbidity has increased [1]. Adverse events (AEs) caused by the administration of many drugs at the same time are therefore a serious concern. These suspected adverse drug reactions (ADRs) due to drug-drug interaction (DDI) can be discovered through post-market drug safety surveillance (PMS). Spontaneous reporting systems (SRSs) are databases used for PMS that include ADR reports and prescription information (e.g, sex, age, date, quantity, etc). By investigating SRS databases using data mining tools, we can identify signals and prevent the potential ADRs induced by DDI. Generally, quantitative DDI signals refer to excessive risk for a combination of two drugs compared with the risks for the individual drugs. However, the criteria used in each method to define signals are different and have various pros and cons.

Several studies have proposed approaches to detect signals indicating drug-drug interactions as well as single-drug adverse reactions. Norén et al. proposed the Ω shrinkage measure to screen for disproportional reporting indicative of suspected drug-drug interaction [2]. Gosho et al. proposed the chi-square statistic as a statistical criterion for detecting drug-drug interaction signals and compared this method with Norén et al.’s Ω shrinkage measure using a simulation study [3].The proportional reporting ratio (PRR) proposed by Evans et al [4] was used to detect signals indicating single-drug reactions, then was extended for drug-drug interactions by Wang et al. [5]. The concomitant signal score (CSS), proposed by Noguchi et al. [6], is an improved detection method using the PRR. Thakrar et al. proposed the additive and multiplicative models [7].

Some studies have reviewed the methodologies [8, 9]. Other studies applied the statistical models to real world data, and then compared the results [10–16]. Only a few studies used simulation to evaluate the performance of a few methods [3]. The main purpose of this study is to assess the performance of various detection methods for DDI through a simulation study. We evaluated the methods in terms of false positive rate and sensitivity in various scenarios. Note that our study exclusively focused on employing frequentist methods for detecting DDI signals. It’s noteworthy to mention that there exists a well-known Bayesian model named Interaction Signal Score (IntSS) [15, 16] for this purpose.

In the next section, we provide an overview of existing methods and explain how the simulation study was implemented. We also introduce the Korea Adverse Event Reporting System (KEARS) data (2017–2019) from the Korea Institute of Drug Safety and Risk Management (KIDS). In the following section, we present the performance of the signal detection algorithms for DDI from the simulation study. The DDI signal detection results using the KEARS data are also shown. We present a discussion in the final section.

## 2. Methods

The signal detection methods are fundamentally based on the observed AE frequencies according to exposure status of two drugs as presented in Table 1. Let *r*_00_ denote the observed reporting rate for the AE in the absence of both Drug 1 and Drug 2. Similarly, *r*_10,_*r*_01_, and *r*_11_ are the observed reporting rate (i) with Drug 1 but not Drug 2, (ii) with Drug 2 but not Drug 1, and (iii) with concomitant use of the two drugs, respectively.

**Table 1:** Observed frequencies (reporting probabilities) table for drug-drug-AE combinations.

Exposure status		AE status		Observed reporting rate
Drug1	Drug2	Yes	No	
No	No	*n*_001_(*p*_00_)	*n*_000_	*r*_00_ = *n*_001_/(*n*_001_ + *n*_000_)
Yes	No	*n*_101_(*p*_10_)	*n*_100_	*r*_10_ = *n*_101_/(*n*_101_ + *n*_100_)
No	Yes	*n*_011_(*p*_01_)	*n*_010_	*r*_01_ = *n*_011_/(*n*_011_ + *n*_010_)
Yes	Yes	*n*_111_(*p*_11_)	*n*_110_	*r*_11_ = *n*_111_/(*n*_111_ + *n*_110_)

### 2.1 Ω shrinkage method

Norén et al. [2] proposed the Ω shrinkage method. The method basically compares the observed reporting rate *r*_11_ with its expected value *E*[*r*_11_] estimated under the assumption that there is no interaction between the two drugs. The estimator *s*_11_ of *E*[*r*_11_] is given as

$$s_{11}=1-\frac{1}{\max\left(\frac{r_{00}}{1-r_{00}},\frac{r_{10}}{1-r_{10}}\right)+\max\left(\frac{r_{00}}{1-r_{00}},\frac{r_{01}}{1-r_{01}}\right)-\frac{r_{00}}{1-r_{00}}+1}.$$

A shrinkage factor is considered to adjust spurious associations due to a very small value of *s*_11_ by reason of generally very rare ADRs by DDI. The Ω shrinkage measure is defined as

$$\Omega =log_{2}\frac{n_{111}+\alpha }{s_{11}n_{11}+\alpha }$$

where *n*_11_ = *n*_111_ + *n*_110_ and *α* is a tuning parameter determining the shrinkage strength. Generally, *α* is set to 0.5. The lower limit of the 95% confidence interval for Ω can be estimated as

$$\Omega_{025}=\Omega -\frac{\phi (0.975)}{\log(2)\sqrt{n_{111}}}$$

where *ϕ*(0.975) is the 97.5th percentile of the standard normal distribution. The criterion Ω_25_ > 0 is used to determine the DDI signal.

### 2.2 Chi-square statistic method

Gosho et al. [3] proposed the chi-square statistic model in order to reduce the false positive rate when events are rare. The measure of the chi-square statistic method χ is the square root of the chi-square test statistic with a correction term to adjust for false positives.


$$\chi =\frac{n_{111}-s_{11}n_{11}-0.5}{\sqrt{s_{11}n_{11}}}$$

The threshold χ > 2 and χ > 2.6 is set for identifying DDI signals. These cutoff values are set based on the 95th and 99th percentiles, respectively, of the chi-square distribution with one degree of freedom.

### 2.3 Proportional reporting ratio (PRR)

The proportional reporting ratio (PRR) proposed by Evans et al. [4] is commonly used in disproportionality analysis to detect adverse event induced by a single drug. The PRR is the ratio of observed reporting rate with and without a drug. The PRR has been extended to drug-drug interactions [5]. First, *PRR*_*D*1_ for Drug1 and *PRR*_*D*1_ for Drug2 are defined as

$$PRR_{D1}=\frac{\left(n_{101}+n_{111}\right)/\left(n_{10}+n_{11}\right)}{\left(n_{001}+n_{011}\right)/\left(n_{00}+n_{01}\right)},$$

$$PRR_{D2}=\frac{\left(n_{011}+n_{111}\right)/\left(n_{01}+n_{11}\right)}{\left(n_{001}+n_{101}\right)/\left(n_{00}+n_{10}\right)}$$

where *n*_10_ = *n*_101_ + *n*_100_, *n*_00_ = *n*_001_ + *n*_000_, and *n*_01_ = *n*_011_ + *n*_010_. The *PRR*_*D*1*D*2_ for concomitant use of Drug1 and Drug2 is defined as

$$PRR_{D1D2}=\frac{n_{111}/n_{11}}{\left(n_{001}+n_{101}+n_{011}\right)/\left(n_{00}+n_{10}+n_{01}\right)}.$$

The lower limit of the 95% confidence interval for the PRR is used to define a signal. It is calculated as

$$PRR_{025}=e^{\text{ln}\;PRR-1.96SD}$$

where SD stands for the standard deviation. The SD for Drug1 is

$SD_{D1}=\sqrt{\frac{1}{n_{101}+n_{111}}-\frac{1}{n_{10}+n_{11}}+\frac{1}{n_{001}+n_{011}}-\frac{1}{n_{00}+n_{01}}}$, the SD for Drug2 is $SD_{D2}=\sqrt{\frac{1}{n_{011}+n_{111}}-\frac{1}{en_{01}+n_{11}}+\frac{1}{n_{001}+n_{101}}-\frac{1}{n_{00}+n_{10}}}$, and the SD for the Drug1-Drug2 pair is $SD_{D1D2}=\sqrt{\frac{1}{n_{111}}-\frac{1}{n_{11}}+\frac{1}{n_{001}+n_{101}+n_{011}}-\frac{1}{n_{00}+n_{10}+n_{01}}}$. The signal detection criterion is to compare the lower limit of the 95% confidence interval for a single drug and drug-drug pairs. If *PRR*_025*D*1*D*2_ > max (*PRR*_025*D*1_, *PRR*_025*D*2_), then the drug pair is considered to be a signal of DDI.

### 2.4 Concomitant signal score (CSS)

The concomitant signal score, proposed by Noguchi et al. [6], was shown to improve the combination risk ratio (CRR) [17], a DDI detection method using PRR. The weakness of CRR is that the lower limit of the 95% CI of *PRR*_*D*1*D*2_ overlaps with the upper limit of the 95% CI of *PRR*_*D*1_ or *PRR*_*D*2_. This is because adverse event reports involving individual drugs are more common than reports concerning the concomitant use of two drugs. The concomitant signal score (CSS) is the ratio of *PRR*_025*D*1*D*2_ and the maximum value between *PRR*_975*D*1_ and *PRR*_975*D*2_.


$$CSS=\frac{PRR_{025D1D2}}{\max\left(PRR_{975D1},PRR_{975D2}\right)}$$

he signal detection criteria are (1) *PRR*_025*D*1*D*2_ > 1 and (2) *CSS* > 1.

### 2.5 Additive model

Thakrar et al. considered both an additive model and a multiplicative model for the detection of DDI signals [7]. The additive model assumes that the risk associated with a drug adds to the background risk. Under the additive assumption, no interaction is established when the excess risk associated with the drug combination is the same as the sum of the excess risks associated with each exposure in the absence of the other. The risk difference is defined as *RD*_*D*1*D*2_ = *p*_11_ –*p*_00_, *RD*_*D*1_ = *p*_10_ –*p*_00_, and *RD*_*D*2_ = *p*_01_ –*p*_00_. When *RD*_*D*1*D*2_ > *RD*_*D*1_ + *RD*_*D*2_, the signal of an interaction is detected. Using the linear probability model in the below, we test for the significance of the interaction term.


$$risk\;of\;event=\alpha +\beta_{1}\;Drug1+\beta_{2}\;Drug2+\beta_{3}\;Drug1*Drug2$$

When *β*_3_ is statistically significantly greater than 0, there is a potential DDI. A positive value of *β*_3_ signifies a positive interaction, namely, an elevated risk for the combination of Drug 1 and Drug 2 compared to that expected based on the individual drugs.

### 2.6 Multiplicative model

The multiplicative model assumes that the risk associated with a drug multiplies with the background risk. The risk ratio is defined as $RR_{D1D2}=p_{11}/p_{00}RR_{D1}=p_{10}/p_{00}$, and $RR_{D2}=p_{01}/p_{00}.$ When $RR_{D1D2}>RR_{D1}\times RR_{D2}$, a signal is detected. To test for the interaction effect based on the multiplicative model, we implement the log linear regression model in the below.


$$log(\;risk\;of\;event\;)=\alpha +\beta_{1}\;Drug\;1+\beta_{2}\;Drug\;2+\beta_{3}\;Drug\;1*Drug2$$

When *β*_3_ is statistically significantly greater than 0, there is a potential DDI. As in the additive model, a positive value of *β*_3_ indicates a positive interaction.

### 2.7 Simulation study

We conducted a simulation study to evaluate the performances of the methods reviewed in the previous section. We considered four different sets of scenarios. Although the basic idea for setting the parameters was adopted from the study by Gosho et al. [3], we created more various situations. While they only considered the additive assumption for an interaction effect, we considered both the additive (scenario sets 1 and 2) and multiplicative (scenario sets 3 and 4) assumptions. Under scenario sets 1 and 3, no interaction was assumed to evaluate the false positive rate, which is the proportion of signals falsely detected for an interaction effect. Under scenario sets 2 and 4, positive interaction effects were created to evaluate sensitivity, which is the proportion of signals correctly detected. In each scenario set, four different scenarios were considered. Four scenarios in set 1 all assumed no interaction based on the additive assumption, that is, $p_{11}-p_{10}-p_{01}+p_{00}=0$. Additionally, scenario (1–1) assumed no effect of each single drug $\left(p_{10}-p_{00}=p_{01}-p_{00}=0\right)$; (1–2) assumed a positive effect of Drug 2 $\left(p_{01}-p_{00}>0,p_{10}-p_{00}=0\right)$; (1–3) assumed the same positive effect for Drugs 1 and 2 $\left(p_{10}-p_{00}=p_{01}-p_{00}>0\right)$; and (1–4) assumed a greater effect of Drug 2 than Drug 1 $\left(p_{01}-p_{00}>p_{10}-p_{00}>0\right)$. Four scenarios in set 2 all assumed a positive interaction effect, i.e., $p_{11}-p_{10}-p_{01}+p_{00}>0.$ Additional settings for each single drug effect for (2–1), (2–2), (2–3), and (2–4) were the same for (1–1), (1–2), (1–3), and (1–4), respectively. Scenario set 3 followed the structure of scenario 1 using the multiplicative assumption. All four scenarios in set 3 assumed no interaction under the multiplicative assumption, i.e., $p_{11}\times p_{00}=p_{10}\times p_{01}$. Similarly, scenario set 4 followed the structure of scenario 2 using the multiplicative assumption. All four scenarios in set 4 assumed $p_{11}\times p_{00}>p_{10}\times p_{01}$. In addition, (3–1) and (4–1) assumed $p_{10}/p_{00}=p_{01}/p_{00}=1$; (3–2) and (4–2) assumed $p_{10}/p_{00}=p_{01}/p_{00}=1$; (3–3) and (4–3) assumed $p_{01}/p_{00}=p_{10}/p_{00}>1$; and (3–4) and (4–4) assumed $p_{01}/p_{00}>p_{10}/p_{00}>1.$

We generated AE count data {*n*_001_,*n*_101_,*n*_011_,*n*_111_} from binomial distributions *B*(*n*_00_,*p*_00_),*B*(*n*_10_,*p*_10_),*B*(*n*_01_,*p*_01_),*B*(*n*_11_,*p*_11_) with $n_{00}=10,000,000,n_{10}=n_{01}=100,000,n_{11}=10,000.$ To prevent the AE count from being zero, *n*_111_ was generated using $B\left(n_{11},p_{11}\right)+1$ following the approach described in the study by Gosho et al. [3]. Different values for {*p*_00_,*p*_10_,*p*_01_,*p*_11_} were used under different scenarios as presented in Tables 2–5. The false positive rate and sensitivity were calculated from 3000 replications.

**Table 2:** False-positive rate of the six methods under simulation scenario 1.

Reporting probability for AE (%)						False positive rate						
						Ω shrinkage method	χ method		PRR	CSS	Additive model	Multiplicative model
*p*_00_	*p*_10_	*p*_01_	*p*_11_	*n*_111_ *	*s*_11_ *n*_11_ *		χ_*thr* = 2_	χ_*thr* = 2.6_				
Scenario 1–1												
0.005	0.005	0.005	0.005	1.5	1.4	0.008	0.013	0.005	0.012	0.003	0.001	0.065
0.05	0.05	0.05	0.05	6.0	7.2	0.007	0.006	0.002	0.017	0.005	0.020	0.055
0.5	0.5	0.5	0.5	50.9	55.3	0.001	0.001	0.000	0.010	0.004	0.029	0.037
Scenario 1–2												
0.001	0.001	0.005	0.005	1.5	0.9	0.031	0.049	0.025	0.013	0.002	0.001	0.003
0.01	0.01	0.05	0.05	6.0	6.5	0.032	0.028	0.008	0.013	0.008	0.016	0.027
0.1	0.1	0.5	0.5	51.0	53.4	0.003	0.003	0.000	0.006	0.002	0.031	0.026
Scenario 1–3												
0.001	0.003	0.003	0.005	1.5	0.8	0.039	0.066	0.029	0.083	0.004	0.001	0.002
0.01	0.03	0.03	0.05	5.9	5.1	0.054	0.048	0.015	0.127	0.051	0.011	0.000
0.02	0.05	0.05	0.08	8.9	8.1	0.055	0.049	0.017	0.114	0.074	0.019	0.000
Scenario 1–4												
0.002	0.003	0.006	0.007	1.7	1.1	0.045	0.058	0.029	0.029	0.005	0.001	0.008
0.002	0.003	0.015	0.016	2.6	2.0	0.046	0.053	0.023	0.023	0.005	0.006	0.002
0.002	0.003	0.03	0.031	4.1	3.6	0.050	0.045	0.017	0.006	0.001	0.009	0.000

* Average values obtained from 3000 replications are presented.

**Table 3:** Sensitivity of the six methods under simulation scenario 2.

Reporting probability for AE (%)						Sensitivity						
						Ω shrinkage method	χ method		PRR	CSS	Additive model	Multiplicative model
*p*_00_	*p*_10_	*p*_01_	*p*_11_	*n*_111_ *	*s*_11_ *n*_11_ *		χ_*thr* = 2_	χ_*thr* = 2.6_				
Scenario 2–1												
0.001	0.001	0.001	0.005	1.5	0.5	0.090	0.205	0.112	0.185	0.013	0.005	0.042
0.01	0.01	0.01	0.05	6.0	2.1	0.581	0.584	0.436	0.567	0.506	0.493	0.764
0.1	0.1	0.1	0.5	51.0	13.2	1.000	1.000	1.000	1.000	1.000	1.000	1.000
Scenario 2–2												
0.001	0.001	0.002	0.005	1.5	0.6	0.075	0.140	0.084	0.094	0.011	0.002	0.024
0.01	0.01	0.02	0.05	6.0	3.0	0.332	0.329	0.218	0.387	0.244	0.252	0.409
0.1	0.1	0.2	0.5	51.1	23.0	0.999	0.999	0.994	1.000	1.000	1.000	1.000
Scenario 2–3												
0.001	0.002	0.002	0.005	1.5	0.6	0.069	0.119	0.069	0.118	0.008	0.001	0.011
0.01	0.02	0.02	0.05	5.9	3.2	0.293	0.284	0.170	0.368	0.185	0.110	0.089
0.1	0.2	0.2	0.5	51.0	28.4	0.986	0.983	0.939	1.000	1.000	0.973	0.409
Scenario 2–4												
0.001	0.002	0.004	0.008	1.8	0.8	0.096	0.126	0.073	0.158	0.011	0.006	0.007
0.01	0.02	0.04	0.08	9.0	5.2	0.348	0.326	0.187	0.295	0.219	0.171	0.026
0.1	0.2	0.4	0.8	81.0	45.2	1.000	1.000	1.000	1.000	1.000	1.000	0.027

* Average values obtained from 3000 replications are presented.

**Table 4:** False-positive rate of the six methods under simulation scenario 3.

Reporting probability for AE (%)						False positive rate						
						Ω shrinkage method	χ method		PRR	CSS	Additive model	Multiplicative model
*p*_00_	*p*_10_	*p*_01_	*p*_11_	*n*_111_ *	*s*_11_ *n*_11_ *		χ_*thr* = 2_	χ_*thr* = 2.6_				
Scenario 3–1												
0.005	0.005	0.005	0.005	1.5	1.3	0.006	0.112	0.003	0.086	0.002	0.000	0.063
0.05	0.05	0.05	0.05	6.0	7.3	0.006	0.004	0.000	0.002	0.004	0.013	0.052
0.5	0.5	0.5	0.5	51.0	54.8	0.000	0.001	0.000	0.000	0.005	0.028	0.033
Scenario 3–2												
0.001	0.001	0.005	0.005	1.5	0.9	0.038	0.061	0.028	0.017	0.003	0.002	0.002
0.01	0.01	0.05	0.05	5.9	6.4	0.024	0.020	0.007	0.014	0.004	0.013	0.028
0.1	0.1	0.5	0.5	51.0	53.0	0.003	0.002	0.000	0.004	0.002	0.035	0.026
Scenario 3–3												
0.001	0.002	0.002	0.004	1.4	0.6	0.049	0.091	0.047	0.062	0.004	0.001	0.006
0.0018	0.003	0.003	0.005	1.5	0.7	0.042	0.075	0.037	0.085	0.006	0.000	0.022
0.018	0.03	0.03	0.05	6.0	4.4	0.113	0.106	0.049	0.119	0.058	0.034	0.047
Scenario 3–4												
0.001875	0.003	0.005	0.008	1.8	0.9	0.069	0.107	0.049	0.137	0.015	0.002	0.018
0.01875	0.03	0.05	0.08	9.0	6.3	0.188	0.170	0.084	0.180	0.117	0.085	0.038
0.04375	0.05	0.07	0.08	9.0	8.3	0.054	0.048	0.015	0.039	0.019	0.026	0.046

* Average values obtained from 3000 replications are presented.

**Table 5:** Sensitivity of the six methods under simulation scenario 4.

Reporting probability for AE (%)						Sensitivity						
						Ω shrinkage method	χ method		PRR	CSS	Additive model	Multiplicative model
*p*_00_	*p*_10_	*p*_01_	*p*_11_	*n*_111_ *	*s*_11_ *n*_11_ *		χ_*thr* = 2_	χ_*thr* = 2.6_				
Scenario 4–1												
0.001	0.001	0.001	0.005	1.5	0.5	0.080	0.213	0.126	0.261	0.013	0.004	0.040
0.01	0.01	0.01	0.05	6.0	2.2	0.538	0.541	0.393	0.565	0.496	0.494	0.765
0.1	0.1	0.1	0.5	50.9	13.0	1.000	1.000	1.000	1.000	1.000	1.000	1.000
Scenario 4–2												
0.001	0.001	0.002	0.005	1.5	0.5	0.079	0.160	0.091	0.113	0.011	0.003	0.019
0.01	0.01	0.02	0.05	5.9	3.0	0.369	0.319	0.212	0.380	0.236	0.250	0.410
0.1	0.1	0.2	0.5	50.9	22.7	1.000	1.000	0.998	1.000	1.000	1.000	1.000
Scenario 4–3												
0.001	0.002	0.002	0.005	1.5	0.6	0.067	0.112	0.061	0.085	0.007	0.001	0.009
0.01	0.02	0.02	0.05	6.0	3.2	0.309	0.296	0.176	0.393	0.198	0.112	0.085
0.1	0.2	0.2	0.5	51.1	28.4	0.988	0.985	0.937	1.000	1.000	0.969	0.398
Scenario 4–4												
0.0008	0.001	0.003	0.005	1.5	0.5	0.084	0.153	0.096	0.100	0.007	0.002	0.003
0.008	0.01	0.03	0.05	6.0	3.8	0.207	0.190	0.103	0.134	0.074	0.092	0.087
0.08	0.1	0.3	0.5	51.0	31.7	0.937	0.922	0.774	0.913	0.891	0.938	0.496

* Average values obtained from 3000 replications are presented.

### 2.8 Korea Adverse Event Reporting Systems (KAERS) data

We applied the six DDI signal detection methods to Korea Adverse Event Reporting System (KAERS) data from the Korea Institute of Drug Safety and Risk Management (KIDS) in 2017–2019. Drug information in KAERS data was documented by the ingredient names with ATC (Anatomical Therapeutic Chemical) codes (https://www.whocc.no/atc_ddd_index/), and AE details were recorded with WHO-ART (WHO Adverse Reaction Terminology) codes (https://www.who-umc.org), eliminating the need for an additional mapping process. We focused on two AEs of QT interval prolongation and hypokalemia. Known interactions for the two AEs were derived from a research report published by the Health Insurance Review and Assessment Service (HIRA) on the adverse event monitoring system, and the list of contraindications of co-medication drugs was derived from KIDS (as of Dec. 28, 2020). In addition, suspected interaction pairs for the same AEs were searched from the combinations of drug-drug-AE that have high frequencies in KAERS data. According to the HIRA research report, it has been reported that co-administration of potassium chloride and spironolactone and co-administration of tactolimus and spironolactone induce hyperkalemia. Potassium chloride is prescribed for potassium-deficiency, electrolyte imbalance, and digitalis poisoning. Spironolactone is a diuretic. Tactolimus is an intensive immunosuppressant agent used to prevent transplant rejection. Co-administration of domperiodone and amiodarone has been reported to cause QT prolongation. Prolongation of the QT interval is associated with an increased risk of development of a potentially lethal cardiac arrhythmia called torsade de pointes, a risk that increases with the administration of a QT-prolonging drugs [18]. Domperidone is a drug that increases gastrointestinal motility and is prescribed for indigestion and vomiting. Amiodarone is an antiarrhythmic drug.

## 3. Results

### 3.1 Simulation study

Tables 2 and 3 present the false positive rate and sensitivity of the six methods under the additive assumption. The false positive rate of the Ω method ranged from 0.001 to 0.055. The false positive rate of the chi-square method with threshold = 2 was similar to that of the Ω method: between 0.001 and 0.066. The false positive rate of the chi-square method with threshold = 2.6 showed the smallest variation, ranging from 0.000 to 0.029, while the PRR method showed a wide range of false positive rate, ranging from 0.006 to 0.127. The Ω method and the chi-square method with threshold = 2 controlled the false positive rate below 0.05 and had high sensitivity in most scenarios. In particular, when the number of events is small (*n*_111_ < 2), the chi-square method has a higher sensitivity than the Ω method. The CSS method showed the lower false positive rate than the Ω method or the chi-square method, but it also showed the lower sensitivity. This may be due to the lower threshold for the measure of the CSS method to reach an interaction. Comparing the additive and the multiplicative model, when there is no effect of each single drug or there is an effect of Drug2 only (scenarios (2–1) and (2–2)), the multiplicative model showed the higher sensitivity than the additive model. On the other hand, when there is an effect of Drug1 and Drug2 (scenarios (2–3) and (2–4)), the additive model showed the higher sensitivity than the multiplicative model.

Comparing scenarios (2–3) and (2–4), most methods except the multiplicative model showed the higher sensitivity in the scenario (2–4). That is, when the difference between the effects of the two drugs is large, the sensitivity increases.

The false positive rate and sensitivity of the six methods under the multiplicative assumption are presented in Tables 4 and 5. In scenario 3 comparing false positive rate, scenarios (3–1) and (3–2) are the same with scenarios (1–1) and (1–2) because they satisfy both the additive assumption and multiplicative assumption. In scenario 4 comparing sensitivity, scenarios (4–1), (4–2), and (4–3) are equivalent to scenarios (2–1), (2–2), and (2–3) because they also satisfy both the additive assumption and multiplicative assumption. It can be shown that satisfying the multiplicative assumption will imply satisfying the additive assumption, thus the additive model has the lower threshold for incidence f to reach an interaction,

The detection signals of the multiplicative model mean stronger interactions than those of the additive model. By comparison, the additive model can detect the DDI signal earlier. The multiplicative model can help determine whether there is stronger evidence of DDI after checking DDI with the additive model.

### 3.2 KAERS data

A total of 1,131,985 reports were taken from the KAERS. Among them, there were 1656 cases of hyperkalemia and 284 cases of QT prolongation. The observed reporting rate for the exposure status of each drug-drug pair was summarized in Table 6. The measure of the six methods for the six drug-drug-AE combinations was summarized in Table 7. A known interaction for QT prolongation, domperidone ‐ amiodarone, was detected as a signal by the Ω method, the chi-square method, the PRR method, the CSS method, and the additive model. The number of reports of domperidone ‐ amiodarone combination was very small (*n*_11_ = 18), but its reporting proportion of the AE was relatively high (4/18). The multiplicative model showed a positive interaction trend for domperidone ‐ amiodarone combination, but it did not reach the statistical significance (p-value = 0.185). For known interactions, only one drug-drug pair (domperidone- amiodarone) was detected. The reason for the reduced sensitivity may be that the number of cases is very small because it is rare to prescribe combinations that are known to have side effects when taken together.

**Table 6:** The proportion of adverse event in the exposure status of each drug-drug pair.

Drug-drug pair	Adverse event	No A, No B	A, No B	No A, B	A and B
Known interaction					
Potassium chloride(A)-spironolactone(B)	Hyperkalemia	1132/1145114	7/2308	505/4813	12/224
Domperidone(A)-amiodarone(B)	QT prolongation	252/1147706	3/2728	25/1533	4/18
Suspected interaction					
Acetylsalicylic acid(A)- polystyrene sulfonate(B)	Hyperkalemia	1541/1124513	43/25783	50/1704	22/459
Acetylsalicylic acid(A)-amiodarone(B)	QT prolongation	254/1124458	1/25976	25/1285	4/266

**Table 7:** The measure of each method applied to drug-drug / adverse events.

Drug-drug / adverse event	Ω shrinkage method (Ω_025_)	χ method	PRR (*PRR*_025*D*1*D*2_)	CSS	Additive (${\hat{\beta }}_{3}$)	Multiplicative (${\hat{\beta }}_{3}$)
Known interaction						
Potassium chloride-spironolactone / Hyperkalemia	-1.780	-2.532	21.607	0.190	-0.053	-1.793
Domperidone-amiodarone / QT prolongation	1.062*	5.741*	382.197*	3.096*	0.205*	1.001
Suspected interaction						
Acetylsalicylic acid- polystyrene sulfonate/ Hyperkalemia	0.072*	2.143*	22.412*	0.735	0.018	0.294
Acetylsalicylic acid-amiodarone / QT prolongation	-1.749	-0.736	23.223	0.188	-0.004	1.511

* indicates statistically significant signals.

Acetylsalicylic acid ‐ polystyrene sulfonate, a suspected interaction for hyperkalemia, was detected as a potential signal by the Ω method, the chi-square method, and the PRR method. The reporting proportion of hyperkalemia for acetylsalicylic acid ‐ polystyrene sulfonate combination was 22/459. While the combination of acetylsalicylic acid and polystyrene sulfonate was not a previously known interaction for hyperkalemia, DDI signals were detected among the suspected combinations that has a large number of AE reports. Only three of the six methods identified DDI signals.

## 4. Discussion

As the number of patients with chronic disease becomes more common, the co-prescription of multiple drugs has increased. Therefore, it has become more important to identify combinations of drugs that have side effects through post-market drug safety surveillance. In this article, we examined statistical methodologies for DDI signal detection. Of the six methods, the Ω shrinkage method and the chi-square method showed the best performance. The Ω shrinkage method and the chi-square method with threshold = 2 controlled the false positive rate below 0.05 and had high sensitivity in most scenarios. The chi-square method was especially effective when there were a very small number of reports for an AE. The chi-square method with threshold = 2.6 and the additive model seemed rather conservative. They rigorously controlled the FPR, but they had lower sensitivity than the Ω shrinkage method and the chi-square method with threshold = 2.

While we undertook a comprehensive simulation study and presented a real data example, there are some limitations in this study. For the simulation study, we assumed the number of cases involving both drugs (*n*_11_) to be 10,000, which is somewhat large. However, when considering the scenario of a very low reporting probability rate for AEs, such as 0.005%, it can be deemed a reasonable value. Nevertheless, it would be beneficial to perform an additional simulation study, assuming smaller values for *n*_11_. We presented the performance of the six methods based on FPR and sensitivity at a fixed threshold for each method. It is worth considering the feasibility of evaluating the methods using multiple possible thresholds through ROC curves and AUCs. For the KAERS data, we evaluated only two AEs of QT interval prolongation and hypokalemia. Additionally, we did not consider the approach involving negative controls for the real data example. As our study primarily focuses on evaluating the six frequentist methods for detecting DDI signals based on a simulation study, we believe it would be more appropriate to present real data examples in a consistent manner. Nevertheless, the utilization of negative controls would be a valuable approach for detecting DDI signals, as well-described in the study by Kontsioti et al. [16].

An important consideration is that spontaneous reporting system databases may result in bias due to underreporting. Since underreporting information is not included in the collected data, it cannot be considered in the analyses. Thus, it is likely to have an impact on DDI signal detection. None of the six methods introduced in this paper adjust for the underreporting bias. We should mention that regardless of the methods used, the underreporting bias and other research limitations of spontaneous reporting databases, such as the Weber effect [19], notoriety effect [20], ripple effec t [20], and masking effect [21], cannot be eliminated [22]. It would be very valuable to develop a DDI signal detection method that can account for the bias. Also, further simulation studies will be needed to investigate the effect of the underreporting bias.

The aforementioned methods have the advantage of being easy to apply and easy to calculate, but the sole use of individual method may not be adequate. It is recommended to use several methods together, rather than just one, to make a reasonable decision. Also, pharmacological mechanisms for drug-drug interactions should be considered. The DDI signal detection considering clinical aspects may further improve the limitations of statistical DDI signal detection methods.
